# Content Analysis of Assessment Tools Used in Post-Stroke Rehabilitation: A Scoping Review with Linkage to the International Classification of Functioning

**DOI:** 10.3390/ijerph22081277

**Published:** 2025-08-15

**Authors:** Maria Heloiza Araujo Silva, Thaissa Hamana de Macedo Dantas, Ana Cecília de Medeiros Araújo, Diego de Sousa Dantas, Maria Isabelle de Araújo Dantas, Beatriz Cristina Medeiros de Lucena, Isabelly Cristina Rodrigues Regalado Moura, Aline Braga Galvão Silveira Fernandes

**Affiliations:** 1 Center for Health Sciences, Federal University of Rio Grande do Norte, Natal 59078-900, Brazil; thaissa_hamana@hotmail.com; 2 Faculty of Health Sciences of Trairi, Federal University of Rio Grande do Norte, Santa Cruz 59200-000, Brazil; cecimaraujo@hotmail.com (A.C.d.M.A.); izabelledantas069@gmail.com (M.I.d.A.D.); bialucena0298@gmail.com (B.C.M.d.L.); isabelly.regalado@ufrn.br (I.C.R.R.M.); linebraga.fisio@gmail.com (A.B.G.S.F.); 3 Center for Health Sciences, Federal University of Pernambuco, Recife 50670-420, Brazil; diego.sdantas@ufpe.br

**Keywords:** cerebrovascular disorders, neurological rehabilitation, surveys and questionnaires, community participation, scoping review

## Abstract

Stroke rehabilitation requires comprehensive assessments aligned with the International Classification of Functioning, Disability, and Health (ICF) biopsychosocial model. Linking assessment tools to the ICF helps integrate this approach by identifying aspects of functioning they address. This study aimed to analyze the content of the most used assessment tools for post-stroke rehabilitation through systematic linkage with the ICF. A scoping review was conducted, including (1) the identification of clinical trials on post-stroke rehabilitation published between 2014 and 2024 in the PubMed, LILACS, SciELO, and PEDro databases to select the most commonly used assessment tools, followed by (2) the ICF linkage methodology to map the most cited tools to the content of ICF categories and domains. From the 897 studies reviewed, 29 tools were identified—21 were newly linked and 8 had pre-existing ICF links. The analysis identified 261 ICF categories: 53% related to Activities, 31% to Body Functions, 15% to Participation, and 1% to Environmental Factors. No tool covered the Body Structure domain. The findings highlight a focus on Activities and Body Functions, reinforcing the need to integrate Participation and Environmental Factors into post-stroke rehabilitation assessments. The results offer an overview of ICF categories covered by each tool, supporting informed decisions in rehabilitation research and practice.

## 1. Introduction

Stroke is the most prevalent neurological disease and the third leading cause of disability worldwide, resulting in functional impairment and a decline in quality of life [1,2,3]. A significant economic burden is associated with post-stroke healthcare maintenance in acute and chronic phases, encompassing hospital costs, rehabilitation, and reduced work productivity [4,5]. Given the residual functional deficits experienced by post-stroke patients and their broader impact beyond physical limitations, it is essential to incorporate tailored rehabilitation interventions into their care [6,7].

In this context, the development and implementation of rehabilitation interventions are recognized as primary tools for disability reduction, positively influencing functioning and quality of life [8]. However, assessment tools used prior to rehabilitation fail to evaluate individuals holistically, considering functioning in a broader sense as proposed by the International Classification of Functioning, Disability, and Health (ICF) [9,10].

The ICF, established by the World Health Organization (WHO), aims to classify individuals’ health status through a biopsychosocial approach. In its conceptual model, functioning results from the interaction between Body Structure, Body Functions, Activities, and Participation, considering Personal and Environmental Factors within the individual’s context [11,12].

Furthermore, the ICF facilitates standardized communication among professionals through information coding and considers the interaction of biopsychosocial factors as indicators of human disability and functioning [13,14]. However, due to its complexity, breadth, and approximately 1450 categories, its application in clinical practice remains challenging [15].

Rehabilitation facilitates disability reduction, so it is essential to incorporate the biopsychosocial model proposed by the ICF during this process. Therefore, assessment tools should encompass the aspects of functioning proposed by the ICF to measure functional status comprehensively [7,15,16,17,18].

Despite numerous tools available for developing robust assessments, gaps remain between what is produced in scientific research and what is implemented in clinical practice [19]. Moreover, assessment tools that solely measure aspects related to Body Function are still widely used, which could be problematic considering the negative implications of stroke on functioning and the context of these patients [20].

To operationalize the use of the ICF in assessing and managing individuals with different health conditions, some strategies have been developed: (1) the development of ICF-based instruments, such as WHODAS 2.0 [21]; (2) the creation of Core Sets [21]; and (3) the linkage of content from existing instruments to ICF categories [20]. These tools ensure that assessment and clinical decision-making consider not only the disease but also its impact on Activities and Participation, providing a broader perspective beyond Body Functions and Structures [22].

Core Sets comprise essential categories for evaluating specific health conditions, guiding and facilitating assessments in clinical and research settings, including rehabilitation [23]. They can be comprehensive, encompassing all relevant ICF categories, or brief, offering a more concise alternative. This approach has clinical value and has been developed and applied in various health conditions, including stroke [24,25].

To improve the comparability of tool information with that outlined in the ICF, utilizing specific linking rules ensures that information is consistently available to serve as a starting point for evidence-based decision-making at all levels of healthcare [26]. These linking rules were developed to facilitate the application of the theoretical model of the ICF, verifying correspondence between assessment procedures such as questionnaires, functional assessment scales, body performance tests, instrumental assessments, and ICF codes or categories [10].

In this context, some studies have already been conducted applying these rules for linking post-stroke assessment instruments to the ICF, such as the Stroke Impact Scale (SIS) [27], Functional Independence Measure (FIM) [28], Visual Analogue Scale (VAS) [29], and Rivermead Mobility Index (RMI) [30]. However, there is a need to analyze other tools geared toward rehabilitation, guiding professionals in conducting comprehensive assessments of post-stroke patients in rehabilitation [31,32,33,34]. Therefore, considering impairments caused by stroke in motor and cognitive function decline contributing to disability [35] and increased rates of anxiety and depression [36], as well as limited social participation, evaluating the patient from a biopsychosocial perspective becomes necessary.

Thus, this study aimed to analyze the content of the most used assessment tools for post-stroke rehabilitation by systematic linkage with the ICF, observing the ICF categories and the domains they covered.

## 2. Materials and Methods

This study is a scoping review conducted in two stages: (1) the identification of assessment tools used in post-stroke rehabilitation through a systematic search and selection process, and (2) the analysis of the selected tools using the ICF linkage methodology to map their content to ICF categories and domains.

### 2.1. Scoping Review

The scoping review aimed to identify the assessment tools most used in clinical research related to post-stroke rehabilitation. It was structured according to the guidelines recommended by the Preferred Reporting Items for Systematic Reviews and Meta-Analysis Extension for Scoping Reviews (PRISMA-ScR): Checklist and Explanation [37] (Supplementary File S1). The project was registered on the Open Science Framework (OSF) and can be accessed via DOI 10.17605/OSF.IO/53VWE or the following link: https://osf.io/53vwe (accessed on 3 May 2025).

We used the PCC (population, concept, context) methodology from the Joanna Briggs Institute to establish the review question and define its eligibility criteria and search strategy. Thus, the population is post-stroke patients, the concept is assessment tools used in clinical trials, and the context is any rehabilitation setting [38].

The review considered studies published between July 2014 and June 2024 (Supplementary Table S1). The inclusion criteria were clinical trials (randomized or non-randomized) addressing post-stroke rehabilitation and written in English, Portuguese, or Spanish. Studies that performed any rehabilitation were included as follows: specialized inpatient, outpatient, or community-based rehabilitation. The exclusion criteria were clinical trial protocols or clinical trials that did not use questionnaires, rating scales, or tests for outcome assessment and did not involve post-stroke patients.

The search strategy followed the three-step process recommended by the Joanna Briggs Institute [39,40]. First, an initial limited search was conducted in PubMed and PEDro to identify relevant keywords and descriptors from titles, abstracts, and keywords of pertinent articles.

Based on this initial analysis, a comprehensive search strategy was developed and adapted for each selected database. The search was conducted in the PubMed, LILACS, SciELO, and PEDRo databases using the following keywords: stroke, rehabilitation, clinical trial, and randomized clinical trial. The search strategies employed were as follows: “stroke AND rehabilitation AND (clinical trial OR randomized controlled trial)” for PubMed, SciELO, and LILACS, and “stroke AND rehabilitation AND (clinical trial* OR randomized controlled trial*)” for PEDro. The keywords were selected according to the PCC methodology, as explained earlier. We chose not to include the keywords “tools”, “scales”, “questionnaires”, or “tests”, as their inclusion excluded many relevant studies. Finally, the reference lists of all included studies were manually screened to identify additional eligible articles.

Two blinded and trained researchers (MI and BC) conducted the initial search and selected studies based on their titles and abstracts. Eligible and potentially eligible articles were read in full text to make the final inclusion decision. A third independent researcher (MH) resolved conflicts.

During data extraction, we collected information from the included studies about the tools used and their frequency of appearance, considering that each tool could be cited more than once in different articles. After extracting the assessment tools mentioned in the included studies, over 500 were identified. Given that this study aimed to conduct the linkage process only with the most frequently cited tools, and considering the impracticality of performing this process with many tools, we chose to select only the most commonly used and cited. This approach aimed to identify ICF content within the most widely used tools and, consequently, those most relevant to post-stroke patients’ rehabilitation research.

Therefore, we established inclusion criteria for the tools included in the linkage process: (1) tools with four or more citations were arranged in ascending order of citations (totaling 98), and (2) tools at or above the 75th percentile were selected.

### 2.2. Linking Process

Before linkage, a search was conducted to verify which tools had already been linked. Tools already linked to the ICF were retained and adequately referenced. The remaining tools underwent the linkage process, which was performed according to the methods proposed by Cieza et al. [41,42], considering updates in the most recent version [26].

According to Alarcos Cieza (2006 and 2016), the linking process of health assessment tools involves mapping items from questionnaires or assessment tools to find relevant content for each item. Then, the relevant content is assigned to ICF categories following a systematic process and rules: direct linking (when the item clearly corresponds to an ICF code), multiple linking (if the item covers more than one ICF concept), or non-linkable (if no relationship exists). The process requires expert consensus and aims to standardize data for research, clinical practice, or public policies, enabling comparisons across different tools.

Two independent researchers (MH and AB), trained in ICF use and coding, performed the linkage and categorization process. A third researcher (TH), experienced in linking tools to the ICF, resolved discrepancies and performed the final category judgments. Cohen’s Kappa Coefficient was used to calculate the level of agreement between the two researchers regarding the ICF categories linked in each tool. This analytical test is commonly used and recommended when performing the linkage methodology. High agreement strengthens confidence that the selected ICF categories are genuinely represented in each tool [20,43]. This coefficient was calculated using Microsoft Excel^®^ 2024.

Finally, descriptive statistical analysis was conducted. Data were expressed in absolute frequency for the presence of ICF categories in the tools considering the Activity and Participation domains (d), Body Functions (s), Body Structures (b), and Environmental Factors (e). Additionally, concepts not covered or defined by the ICF were also considered based on the methods proposed by Cieza et al. [20].

## 3. Results

Regarding the scoping review, 8926 studies were initially identified. Of these, 897 met the inclusion criteria. From a total of 586 assessment tools identified, 29 were ultimately selected as they fulfilled the predefined selection criteria.

**Figure 1:**
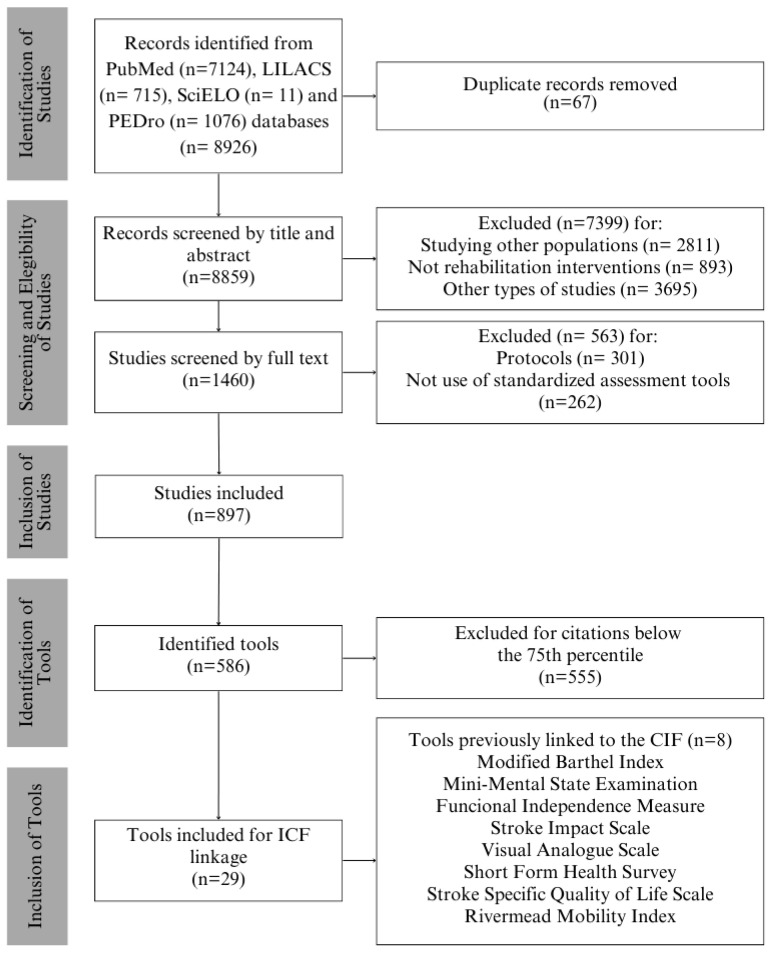
Identification, screening, and inclusion of studies and assessment tools.

Those tools for which linking was already available in the literature were included in our results and appropriately referenced. The questionnaires that already had a link to the ICF were as follows: the Functional Independent Measure (FIM) [28], Modified Barthel Index (MBI) [25], Modified Rankin Scale (mRS) [44], Short Form-36 Health Survey (SF-36) [29], Stroke Impact Scale (SIS) [27], Visual Analogue Scale (VAS) [29], Mini-Mental State Examination (MMSE) [45], and Rivermead Mobility Index (RMI) [30]. The mRS already had a link; however, new linking was performed due to researchers’ disagreements with the data available. Therefore, among the 29 included tools, the previous linking of eight was considered, and the linking of 21 was conducted anew. Figure 1 outlines the search procedures, data extraction, and tool selection. Table 1 presents the selected tools.

Considering the links to ICF categories, 20 of the 29 tools include Body Functions categories, 23 Activities categories, 6 Participation categories, and only 1 evaluates Environmental Factors. Five concurrently assessed Body Functions, Activity, and Participation, and only one analyzed Body Functions, Activity, Participation, and Environmental Factors simultaneously. Table 2 presents the distribution of categories and their percentage for ICF domains in each tool.

From all the tools included, 261 ICF categories were identified. Most corresponded to Activities (53%) and Body Functions (31%). Only 15% of the categories represented the Participation domain. Environmental Factors (1%) were observed less. No tool addressed the Body Structures domain. Six concepts referred to nd (not definable), nc (not contained), nd-gh (not definable—general health), nd-ph (not definable—physical health), nd-mh (not definable—mental health), and nc-hc (not contained—health condition not covered).

The level of agreement was calculated using Kappa for each tool, yielding the following values: 10 MWT:1; ARAT: 0.738; BBT: 1; BBS: 0.785; FAC: 0.799; FMA: 0.972; MAL: 0.734; MI: 1; MAS: 1; MoCA: 0.725; MRC: 0.873; mRS: 1; NIHSS: 0.893; TC6: 1; TUG: 1; WMFT: 0.714; EQ-5D: 1; TIS: 1; BRS: 0.879; BRPE: 1; and HADS: 0.729. The values obtained indicate good inter-rater agreement.

## 4. Discussion

This study aimed to analyze the content of the most used assessment tools for post-stroke rehabilitation by systematic linkage with the ICF, highlighting the ICF categories and domains covered by the instruments. Of the twenty-nine tools included (eight with prior linkage to the ICF), most ICF categories were associated with the Activities domain, followed by Body Functions, Participation, and Environmental Factors. None of the tools assessed Body Structures. Notably, of the five most used assessments, four primarily evaluate Body Functions, and considering the ten most frequently cited, none explicitly assesses Participation.

The most significant number of linked categories involved Activities and Body Functions. Since functioning is defined as a positive interaction between ICF domains, evaluating these two domains is essential [47]. The emphasis on Activities is understandable, as they are closely linked to autonomy in daily life, where most patients experience significant deficits in performing Activities of Daily Living (ADL) [48], and ultimately determine a person’s level of disability [49]. The focus on Activities in research might shift attention away from Body Functions, which were previously more prominent in studies and clinical practice [50]. However, this emphasis on Activities and Body Functions may result in a fragmented view of functioning and limit the effectiveness of rehabilitation strategies.

The limitation in assessing Body Structures may not be considered a problem. Assessing Body Structures requires identifying structural alterations in anatomical components. According to the ICF, these alterations include total or partial absence (e.g., limb amputation), qualitative changes (e.g., tissue degeneration), abnormal positioning (e.g., scoliosis), variations in size or quantity (e.g., muscle atrophy/hypertrophy), and discontinuity (e.g., fractures). Following the linkage rules by Cieza et al. [42,43], categories are only linked when such aspects are explicitly assessed, not simply mentioned. Given these classifications of Body Structure alterations, rehabilitation assessment tools typically do not evaluate this domain directly. Such evaluations usually require imaging studies or other specialized examinations.

Participation was also rarely evaluated, despite its relevance in capturing the patient’s reintegration into daily life, community roles, and overall social functioning [51]. Interestingly, none of the linked tools primarily or exclusively assessed this component. This gap suggests that post-stroke research and rehabilitation still focus on impairment-related outcomes rather than on patient-centered perspectives, which include social reintegration. Although tools like the Functional Autonomy Measurement System (SMAF) [52], Reintegration to Normal Living Index (RNLI) [53], Frenchay Activities Index (FAI) [54], Utrecht Scale for Evaluation of Rehabilitation-Participation (USER-Participation) [55], and Personal Care Participation Assessment and Resource Tool (PC-part) [52] measure Participation, their usage was limited, with only one or two citations for RNLI, SMAF, and FAI. Consequently, these tools were not included in the study analysis due to insufficient representation.

A potential explanation for the lack of Participation-focused tools in clinical trials is the perceived difficulty of measuring and rehabilitating a subjective and multidimensional construct, influenced by contextual and Environmental Factors. The low frequency of citations for Participation assessment tools highlights that this outcome has been underrepresented and has yet to receive the necessary attention [56,57]. Despite its relevance as a primary target for interventions in chronically ill patients [58], there is a lack of awareness regarding the importance of Participation as a rehabilitation outcome. As such, it should be assessed routinely and systematically. Clinical trials must include at least one outcome related to Participation, ideally including specific tools for this assessment.

Environmental Factors were assessed in only one of the 29 tools included, the Modified Rankin Scale (mRS). However, factors like support from family and friends, accessibility to products and technologies, and adaptations for performing ADL can significantly influence recovery and functioning [59,60]. Their underrepresentation highlights a focus on impairments and limitations, with interventions rarely addressing external barriers or facilitators. Incorporating Environmental Factors into assessments can help clinicians identify external influences on recovery and tailor interventions, as the quality of life can also be affected by the interplay between individuals and their environment [61,62]. Future research should focus on operationalizing environmental factor assessments in clinical trials and rehabilitation, as current tools primarily address Participation, not Environmental Factors.

Notably, the Modified Rankin Scale (mRS) was the only tool to include categories from all four ICF components and had the highest number of linked categories, including 22 related to Participation. Furthermore, it was the only assessment tool representing Environmental Factors among those categorized. Its conciseness, combined with a broad scope of content, suggests that it may be particularly useful in clinical practice, especially in busy services where time constraints often limit comprehensive assessment.

Considering patient-centered care, incorporating the ICF Core Sets into clinical practice is essential [63]. The Brief and Comprehensive Core Sets for stroke offer a structured and contextual framework for assessing functioning concerning their contextual interactions [13,64]. Standardized frameworks, such as these ICF Core Sets for stroke, should be further explored to facilitate comprehensive assessments that include Participation and Environmental Factors, mainly the comprehensive version. In this study, at least one of the tools analyzed covered most categories from the Brief Core Set [13]. Exceptions included brain and upper limb structures (s110 and s730) and Environmental Factors related to health professionals and service systems (e355 and e580). Notably, the Brief Core Set lacks specific Participation categories, which may inadvertently discourage professionals from addressing this domain when using the Core Set as their primary reference.

Despite the well-established biopsychosocial model, the most commonly used tools still focus on impairments and activity limitations, neglecting key aspects like Participation and Environmental Factors. Without adequately assessing these dimensions, rehabilitation strategies risk being incomplete and misaligned with patients’ lived experiences. Functioning and disability are shaped by the interaction of Body Functions and Structures, Activities and Participation, and contextual factors [65]. Thus, each aspect must be considered to assess functioning and disability in a biopsychosocial manner [66]. Adopting a biopsychosocial approach is vital for understanding patients’ needs and enhancing treatment effectiveness and efficiency through adherence and identification with treatment. Future research must move beyond this restricted focus to advance evidence-based care and actively integrate tools that assess broader contextual factors and contribute to holistic, patient-centered interventions that truly support post-stroke recovery.

The results of this study provide valuable insights that can enhance the clinical assessment of post-stroke patients, helping rehabilitation professionals choose tools aligned with the ICF categories. By clarifying the scope of these tools, clinicians can make more informed decisions, ensuring a holistic evaluation of functional status. The findings highlight the need for instruments that incorporate not just impairments and activity limitations but also Participation and Environmental Factors, which are critical for shaping rehabilitation outcomes. Integrating these factors into clinical practice can lead to more personalized and context-sensitive interventions, ultimately improving patient recovery and advancing evidence-based rehabilitation.

As the limitations of this study, we acknowledge that the selection criteria aimed to ensure a focus on widely applied tools in rehabilitation research, which may have led to the exclusion of potentially relevant tools less frequently cited in the literature. The 10-year review period can also be considered a limitation. However, this decision was based on recognizing that the adoption of ICF principles is relatively recent. Expanding the search period could have resulted in the inclusion of older tools that are no longer widely used or the exclusion of recent instruments that incorporate these aspects. Additionally, while we recognize the importance of EMBASE, operational constraints led to its exclusion from our search strategy. However, we believe that our focus on freely accessible databases was sufficient to capture key evidence, as our study applied a citation-based criterion to retain the most frequently cited tools, minimizing the impact of this limitation. Future studies should consider the strategies to include, particularly tools that assess Participation and Environmental Factors, which remain underrepresented in current assessments.

## 5. Conclusions

This study analyzed the content of the most widely used assessment tools in post-stroke rehabilitation through systematic linkage with the ICF, identifying the domains and categories they cover. Among the 29 most cited tools, 261 ICF categories were mapped, with a predominance of those related to Activities and Body Functions. In contrast, Participation and Environmental Factors were underrepresented, and no tool assessed Body Structures.

The results provide a comprehensive overview of the ICF domains covered by each tool, facilitating access to this information and supporting more informed decision-making in rehabilitation research and clinical practice.

Despite the availability of numerous assessment instruments, the emphasis on Activities and Body Functions may limit a genuinely biopsychosocial approach to rehabilitation. Future research should prioritize tools that integrate Participation and Environmental Factors to generate more substantial scientific evidence and guide assessments and interventions that address these essential aspects of post-stroke recovery.

## Figures and Tables

**Table 1:** List of assessment tools included for linkage with ICF categories, with their number of citations. Previously linked tools were included with references to the original studies.

Tools	Number of Citations
Fugl Meyer Assessment (FMA) *	375
Modified Barthel Index (MBI) [25]	209
Modified Ashworth scale (MAS)	206
Mini-Mental State Examination (MMSE) [45]	158
National Institute of Health Stroke Scale (NHISS)	128
Berg Balance Scale (BBS)	120
Timed Up and Go (TUG)	93
10-Meter Walk Test (10 MWT)	89
Modified Rankin Scale (mRS)	86
Action Research Arm Test (ARAT)	79
Montreal Cognitive Assessment (MoCA)	78
Wolf Motor Function Test (WMFT)	74
6-Minute Walk Test (6 MWT)	71
Functional Ambulation Categories (FAC)	61
Stroke Impact Scale (SIS) [27]	61
Functional Independence Measure (FIM) [28]	60
Box and Block (BBT)	58
Motor Activity Log (MAL)	45
Visual Analogue Scale (VAS) [29]	40
Brunnstrom Recovery Stage (BRS)	38
Medical Research Council (MRC)	30
Motricity Index (MI)	30
Short Form-36 Health Survey (SF-36)	28
Hospital Anxiety and Depression Scale (HADS)	28
European Quality of Life 5-Dimension (EQ-5D)	27
Stroke Specific Quality of Life Scale (SSQoL) [46]	25
Trunk Impairment Scale (TIS)	23
Borg Rating of Perceived Exertion (RPE)	22
Rivermead Mobility Index (RMI) [30]	21

* FMA (183 citations), FMA-Upper Extremity (149 citations), and FMA-Lower Extremity (43 citations).

**Table 2:** Overview of the tools in alphabetical order considering the distribution of categories and their percentage for each ICF domain.

Tool	Body Functions	%	Activities and Participation	%	Environmental Factors	%	Others
10-Meter Walk Test (10 WMT)		0%	d4500; d465	100%		0%	
Action Research Arm Test (ARAT)		0%	d4300; d4301; d4400; d4401; d4402; d4453; d4458	100%		0%	
Box and Block Test (BBT)		0%	d4301; d4400; d4403	100%		0%	
Berg Balance BERG (BERG)		0%	d4103; d4104; d4105; d4106; d4153; d4154; d4200; d429; d4400	100%		0%	
EuroQol (EQ-5D)	b280	11%	d450, d510; d540; d859; d839; d640; d760; d920	34% 55%		0%	nd-gh; nc-hc; pf
Functional Ambulation Categories (FAC)		0%	d4500; d4502; d4551; d465	100%		0%	
Fugl Meyer Assessment (FMA)	b28016; b260; b265; b7100; b7300; b7500; b7600; b7602; b7651	64%	d4106; d4153; d4154; d4400; d4401	36%		0%	
Motor Activity Log (MAL)	b7600	5%	d170; d2100; d2101; d4104; d4300; d4301; d4402; d4450; d4451; d4453; d5100; d5102; d5200; d5201; d5202; d5402; d5403; d550; d6402	90% 5%		0%	
Motricity Index (MI)	b7300	50%	d4400	50%		0%	
Modified Ashworth Scale (MAS)	b735	100%		0%		0%	
Montreal Cognitive Assessment (MoCA)	b1400; b1440; b1441; b1442; b1560; b1561; b1565; b164; b1640; b167; b16700; b16710; b1720	100%		0%		0%	
Medical Research Council (MRC) *	b1670; b7300; b7600	75%	d210	25%		0%	
National Institutes of Health Stroke Scale (NIHSS)	b1100; b1140; b11420; b156; b167; b1670; b2101; b2703; b320; b3300; b7300; b7301; b7401; b7600; b7602	88%	d210	6%		0%	
6-Minute Walk Test (6 MWT)	b4202; b455	67%	d4500	33%		0%	
Time Up and Go (TUG)		0%	d4103; d4104; d4108; d4500	100%		0%	
Wolf Motor Function Test (WFMT)	b7301; b7600; b7602	75%	d4300; d4301; d4400; d4401; d4402; d4450; d4451; d4452; d4453	25%		0%	
Functional Independence Measure (FIM)	b144; b525; b620	18%	d175; d310; d330; d410; d4500; d4551; d465; d510; d520; d530; d540; d550; d560; d710	76% 6%		0%	
Mini-Mental State Examination (MMSE)		83%	d210	17%		0%	
Modified Barthel Index (MBI)		0%	d410; d420; d450; d455; d5; d510; d530; d540; d550; d560	100%		0%	
Modified Rankin Scale (mRS)	b210; b270; b3300; b5105; b75; b755; b760	14%	d140; d170; d450; d465; d475; d489; d4608; d5100; d5108; d520; d5201; d5202; d530; d5400; d5401; d550; d599; d640; d6401; d6409; d6060; d860; d6300; d839; d8450; d870; d85; d859; d9; d920; d9200; d9201; d9202; d9203; d9204; d9205; d930; d7500; d760	35% 45%	e310, e320; e399	6%	nd; nc
Short Form-36 Health Survey (SF-36)	b1300; b1263; b1265; b152; b280	20%	d230; d4; d4101; d4102; d4105; d430; d4300; d4500; d4501; d4551; d4552; d5; d510; d540; d6402; d6403; d750; d850; d9201; d9205	56% 24%		0%	nd-gh; nd-ph; nd-mh; nc-hc
Stroke Impact Scale (SIS)	b525; b620	11%	d410; d4153; d4154; d420; d430; d450; d4508; d4550; d4551; d5101; d530; d5300; d5301; d5400; d6200; d640	78% 11%		0%	
Visual Analogue Scale (VAS)	b280	100%		0%		0%	

* MRC Comprehension Score. The Activities and Participation domain was subdivided into “d1 to d5” for “Activities” and “d6 to d9” for “Participation”, which is underlined. Body Structures were not covered by any tool. nc: not contained; nc-hc: not contained—health condition not covered; nd: not definable; nd-gh: not definable—general health; nd-ph: not definable—physical health; nd-mh: not definable—mental health; pf: Personal Factors.

## Data Availability

The raw data supporting the conclusions of this article will be made available by the authors on request.

## Supplementary Materials

The following supporting information can be downloaded at: https://www.mdpi.com/article/10.3390/ijerph22081277/s1, Table S1: presenting the search strategy adopted in each database and the number of studies retrieved from 2014 to 2024.; File S1 Preferred Reporting Items for Systematic reviews and Meta-Analyses extension for Scoping Reviews (PRISMA-ScR) Checklist.
