# Supplementary material for: Content Analysis of Assessment Tools Used in Post-Stroke Rehabilitation: A Scoping Review with Linkage to the International Classification of Functioning
Source: Int J Environ Res Public Health. 2025 Aug 15;22(8):1277. doi: 10.3390/ijerph22081277 (PMC12386398; doi:10.3390/ijerph22081277)
Supplement: Supplementary file 1 [file ijerph-22-01277-s001.zip › Supplementary Material.pdf]

Table S1 presenting the search strategy adopted in each database and the number of studies retrieved from 2014 to 2024.

Databases	PubMed	Lilacs	Scielo	PEDro	
Keywords	stroke AND rehabilitation AND (clinical trial OR randomized controlled trial)	(stroke) AND (rehabilitation) AND (clinical trial) OR (randomized clinical trial)	(stroke AND rehabilitation) AND (clinical trial OR randomized clinical trial)	stroke AND rehabilitation *Filter: "Clinical trial" and "New records added since: 01/07/2014"	
Search Period	2014-2024				Total number of articles
Number of Results	7.124	715	11	1.076	8.926
Eligible for title and abstract screening after duplicate removal	7.089	706	11	1.053	8.859
Excluded after title and abstract screening	5.723	655	11	1.010	7.399
Eligible for full-text review	1.366	51	0	43	1.460
Excluded after full-text review	520	10	0	33	563

Final included studies	846	41	0	10	897
